# Thermoreversible crystallization-driven aggregation of diblock copolymer nanoparticles in mineral oil†
†Electronic supplementary information (ESI) available: Assigned ^1^H NMR spectra; digital images of 20% w/w dispersions; TEM images obtained at 20 °C; overlaid SAXS patterns; WAXS patterns; SAXS models used. See DOI: 10.1039/c8sc00762d


**DOI:** 10.1039/c8sc00762d

**Published:** 2018-04-02

**Authors:** Matthew J. Derry, Oleksandr O. Mykhaylyk, Anthony J. Ryan, Steven P. Armes

**Affiliations:** a Department of Chemistry , The University of Sheffield , Dainton Building, Brook Hill , Sheffield , South Yorkshire S3 7HF , UK . Email: m.derry@sheffield.ac.uk ; Email: s.p.armes@sheffield.ac.uk ; Email: a.ryan@sheffield.ac.uk

## Abstract

A poly(behenyl methacrylate)_37_ (PBeMA_37_) macromolecular chain transfer agent is utilized for the reversible addition–fragmentation chain transfer (RAFT) dispersion polymerization of benzyl methacrylate (BzMA) directly in mineral oil at 90 °C. Polymerization-induced self-assembly (PISA) occurs under these conditions, yielding a series of sterically-stabilized PBeMA_37_–PBzMA_*x*_ diblock copolymer spheres of tunable diameter as confirmed by dynamic light scattering (DLS) and transmission electron microscopy (TEM) studies. Rheological studies indicate that a relatively transparent, free-flowing, concentrated dispersion of non-interacting 32 nm PBeMA_37_–PBzMA_100_ spheres at 50 °C forms a turbid, paste-like dispersion on cooling to 20 °C. Turbidimetry and differential scanning calorimetry (DSC) studies conducted on solutions of PBeMA_37_ homopolymer in mineral oil suggest that this switchable colloidal stability is linked to crystallization-induced phase separation exhibited by this stabilizer block. Indeed, variable-temperature small-angle X-ray scattering (SAXS) indicates that a loose mass fractal network of strongly interacting spheres is formed on cooling to 20 °C, which accounts for this thermoreversible sol–gel transition. Moreover, SAXS, DSC and wide-angle X-ray scattering (WAXS) analyses indicate that the behenyl (C_22_H_45_) side-chains first form crystalline domains comprising adjacent stabilizer chains within individual spherical nanoparticles, with subsequent crystallization between neighboring nanoparticles leading to the formation of the mass fractal aggregates.

## Abstract 2

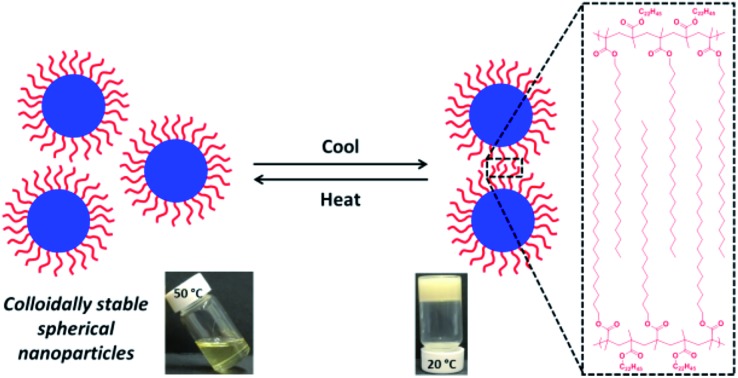
Poly(behenyl methacrylate)-stabilized diblock copolymer nanoparticles are prepared in mineral oil *via* polymerization-induced self-assembly. Such nanoparticles are colloidally stable at 50 °C but undergo reversible crystallization-driven aggregation at 25 °C.

## Introduction

Over the past 20 years or so, there has been considerable and growing interest in crystallization-driven block copolymer self-assembly (CDSA). Manners and co-workers reported the first example of CDSA, which involved the formation of cylindrical micelles by polydimethylsiloxane–poly(ferrocenyldimethylsilane) (PDMS–PFDMS) diblock copolymers in *n*-hexane.^1^ It was shown that the crystallization of the core-forming PFDMS block is solely responsible for the self-assembly behavior.^2^ Since this seminal work, the same group have utilized both ferrocenyl^3^–^10^ and thiophene-based^11^–^15^ diblock copolymers to generate rod-like cylindrical micelles with remarkably well-defined dimensions. In related work, Dove and co-workers have explored the CDSA of block copolymers comprising crystalline poly(l-lactide) (PLLA) cores.^16^–^20^ More recently, judicious solvent selection enabled the formation of relatively uniform micrometer-sized diamond-shaped lamellae from poly(*N*,*N*-dimethylacrylamide)–poly(l-lactide) (PDMAC–PLLA) diblock copolymers.^21^ Furthermore, PDMAC–PLLA–PDMAC triblock copolymers formed either diamond-shaped lamellae or cylindrical micelles of varying length in dilute methanolic solution.^22^ Similarly, Lecommandoux and co-workers^23^ reported the 1D fusion of spherical diblock copolymer micelles to form fibres on prolonged heating at 65 °C in water, with wide-angle X-ray scattering (WAXS) studies indicating the gradual formation of crystalline cores. There have also been several reports of CDSA formulations utilizing poly(ε-caprolactone) core-forming blocks.^24^–^26^

Prior to the development of CDSA, Richter and co-workers^27^ demonstrated that poly(ethylene-propylene)–polyethylene diblock copolymers in *n*-decane form crystalline lamellar structures on cooling from 70 °C to ambient temperature. Later, Xu *et al.*^28^,^29^ utilized small-angle X-ray scattering (SAXS) to demonstrate that diblock copolymer micelles with crystalline cores can form higher order lamellar structures. For example, cooling solutions of poly(ethylene oxide)–poly(butylene oxide) diblock copolymers in *n*-hexane caused spherical micelles to become highly anisotropic owing to crystallization of the core-forming poly(ethylene oxide) chains.^28^ For diblock copolymers with relatively short corona blocks, unfavorable interactions between the crystalline micelle cores and the solvent led to particle aggregation, with pastes being formed even at relatively low copolymer concentrations (<1.0% w/v). In contrast, longer corona blocks prevented aggregation and such dispersions remained free-flowing at copolymer concentrations of up to 10% w/v. Similarly, Schurtenberger and co-workers^30^ observed a reversible spherical micelle-to-lamellar transition for poly(ethylene oxide)–polybutadiene nanoparticles in ethanol on cooling from 60 °C to 20 °C; concomitant WAXS studies confirmed crystallization of poly(ethylene oxide) stabilizer (or corona) blocks.

Recently, many research groups have shown that polymerization-induced self-assembly (PISA)^31^–^35^ is a versatile and efficient method for preparing a wide range of functional diblock copolymer nano-objects (typically spheres, worms or vesicles) in water,^36^–^40^ lower alcohols^41^–^45^ or non-polar solvents,^46^–^52^ as well as various solvent mixtures.^53^–^61^ Such PISA syntheses provide convenient access to new red blood cell cryopreservation protocols,^62^ sterilizable hydrogels for 3D cell growth,^63^ novel stem cell storage media,^64^ model Pickering emulsifiers,^65^–^70^ controlled release of encapsulated payloads^71^ and hitherto unknown high-temperature oil-thickening mechanisms.^72^ Of particular relevance to the present work, reversible addition–fragmentation chain transfer (RAFT) dispersion polymerization enables the PISA synthesis of well-defined spherical nanoparticles of tunable diameter.^73^–^77^ This is typically achieved by systematically varying the target degree of polymerization (DP) of the solvophobic block while utilizing a sufficiently long solvophilic macromolecular chain transfer agent (macro-CTA).^41^,^75^,^76^

**Scheme 1:**
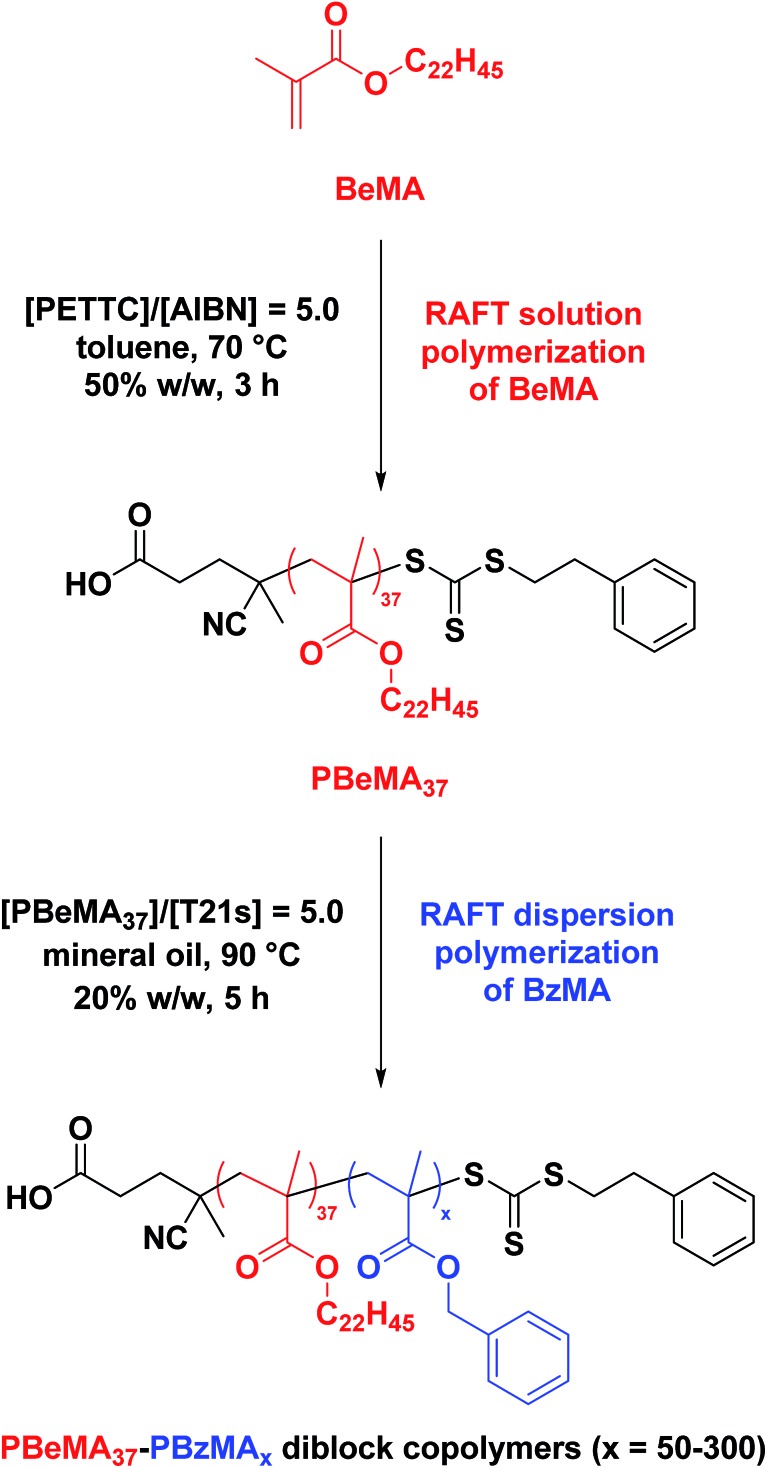
Synthesis of a near-monodisperse poly(behenyl methacrylate) (PBeMA) macro-CTA *via* RAFT solution polymerization of behenyl methacrylate (BeMA) in toluene using 4-cyano-4-(2-phenylethane sulfanylthiocarbonyl)sulfanylpentanoic acid (PETTC) at 70 °C, followed by the RAFT dispersion polymerization of benzyl methacrylate (BzMA) in mineral oil at 90 °C.

In the present study, a series of colloidally-stable poly(behenyl methacrylate)–poly(benzyl methacrylate) (PBeMA–PBzMA) spherical nanoparticles are synthesized *via* RAFT dispersion polymerization of benzyl methacrylate in mineral oil at 90 °C (see Scheme 1). This industrially-sourced solvent was utilized to highlight the versatility and relevance of PISA formulations. We show that the PBeMA stabilizer chains exhibit crystallization-driven aggregation on cooling to 20 °C, which leads to the formation of a macroscopic paste. This colloidal (in)-stability is demonstrated to be thermoreversible and has been characterized using turbidimetry, differential scanning calorimetry (DSC), SAXS, WAXS and rheology.

## Experimental

### Materials

A 4 cSt American Petroleum Institute (API) group III mineral oil and behenyl methacrylate (BeMA, >99%) were kindly provided by The Lubrizol Corporation Ltd (Hazelwood, Derbyshire, UK). 4-Cyano-4-(2-phenylethane sulfanylthiocarbonyl)sulfanylpentanoic acid (PETTC, >99%) was synthesized according to the literature.^69^*Tert*-butyl peroxy-2-ethylhexanoate (T21s, >97%) initiator was purchased from AkzoNobel (The Netherlands), THF and toluene were purchased from Fisher Scientific (UK), CDCl_3_ was purchased from VWR International (UK), CD_2_Cl_2_ was purchased from Goss Scientific (UK) and all other materials were purchased from Sigma-Aldrich (UK) and were used as received.

### Synthesis of poly(behenyl methacrylate) (PBeMA) macromolecular chain transfer agent (macro-CTA) *via* RAFT solution polymerization

A PBeMA_37_ macro-CTA was synthesized as follows: a 250 mL round-bottomed flask was charged with behenyl methacrylate (BeMA; 23.8 g, 60.3 mmol), PETTC (410 mg, 1.21 mmol; target PBeMA DP = 50), 2,2′-azobisisobutyronitrile (AIBN, 98%; 39.6 mg, 241 μmol; [PETTC]/[AIBN] molar ratio = 5.0) and toluene (24.2 g). The reaction solution was purged with nitrogen and placed in a pre-heated oil bath at 70 °C for 3 h. The resulting PBeMA (BeMA conversion = 57%; *M*_n_ = 12 400 g mol^–1^, *M*_w_/*M*_n_ = 1.18) was purified by twice precipitating into excess 2-propanol. The mean degree of polymerization (DP) of this macro-CTA was calculated to be 37 (CTA efficiency = [(target DP × monomer conversion)/(actual DP) = 77%]) using ^1^H NMR spectroscopy by comparing the integrated signals corresponding to the five aromatic protons at 7.0–7.5 ppm with that assigned to the two oxymethylene protons of PBeMA at 3.4–4.2 ppm (see Fig. S1a†).

### Synthesis of poly(behenyl methacrylate)–poly(benzyl methacrylate) (PBeMA–PBzMA) diblock copolymer nanoparticles *via* RAFT dispersion polymerization

A typical RAFT dispersion polymerization synthesis of targeted PBeMA_37_–PBzMA_100_ diblock copolymer nanoparticles at 20% w/w solids was conducted as follows: benzyl methacrylate (BzMA; 0.27 g, 1.54 mmol), T21s initiator (666 μg; 3.08 μmol; dissolved at 10% v/v in mineral oil) and PBeMA_37_ macro-CTA (0.23 g; 15.4 μmol; [macro-CTA]/[initiator] molar ratio = 5.0) were co-dissolved in mineral oil (2.00 g). The reaction mixture was purged with nitrogen for 30 min and the deoxygenated solution was then placed in a pre-heated oil bath at 90 °C for 5 h (final BzMA conversion = 99%; *M*_n_ = 22 700 g mol^–1^; *M*_w_/*M*_n_ = 1.15).

### Gel permeation chromatography (GPC)

Molecular weight distributions were assessed by GPC using THF eluent. The THF GPC set-up comprised two 5 μm (30 cm) mixed C columns and a WellChrom K-2301 refractive index detector operating at a wavelength of 950 ± 30 nm. The mobile phase contained 2.0% v/v triethylamine and 0.05% w/v butylhydroxytoluene, and the flow rate was 1.0 mL min^–1^. A series of ten near-monodisperse poly(methyl methacrylate) standards (*M*_p_ values ranging from 645 to 2 480 000 g mol^–1^) were used for calibration.

### ^1^H nuclear magnetic resonance (NMR) spectroscopy

Homopolymer and copolymer spectra were recorded in either CD_2_Cl_2_ or CDCl_3_ using a Bruker AV1-400 MHz spectrometer. Typically 64 scans were averaged per spectrum.

### Dynamic light scattering (DLS)

DLS studies were performed using a Zetasizer NanoZS instrument (Malvern Instruments, UK) at a fixed scattering angle of 173°. Copolymer dispersions were diluted to 0.10% w/w using *n*-dodecane prior to light scattering studies. The intensity-average diameter and polydispersity index [PDI = (standard deviation/intensity-average diameter)^2^] of the diblock copolymer nanoparticles were calculated by cumulant analysis of the experimental correlation function using Dispersion Technology Software version 6.20, accounting for the temperature-dependent viscosity of the *n*-dodecane. Data were averaged over thirty runs each of thirty seconds duration.

### Transmission electron microscopy (TEM)

TEM studies were conducted using a Philips CM 100 instrument operating at 100 kV and equipped with a Gatan 1 k CCD camera. Diluted diblock copolymer dispersions (0.10% w/w) were placed as droplets on carbon-coated copper grids, allowed to dry and then exposed to ruthenium(viii) oxide vapor for 7 min at 20 °C prior to analysis. This heavy metal compound acted as a positive stain for the core-forming PBzMA block in order to improve electron contrast. The ruthenium(viii) oxide was prepared as follows. Ruthenium(iv) oxide (0.30 g) was added to water (50 g) to form a black slurry; addition of sodium periodate (2.0 g) with stirring produced a yellow solution of ruthenium(viii) oxide within 1 min.^78^

### Turbidimetry

Turbidimetry measurements were performed on an unstirred 1.0% w/w solution (or dispersion) using a Shimadzu UV-1800 spectrophotometer equipped with a twin Peltier temperature controller (DBS Analytical instruments, Italy). Data were recorded at 650 nm during cooling (50 °C to 20 °C) and heating (20 °C to 50 °C) steps, allowing 5 min for thermal equilibration at each temperature. Data were averaged over three measurements.

### Differential scanning calorimetry (DSC)

DSC studies were performed using a TA Instruments Discovery DSC instrument equipped with TZero low-mass aluminium pans. Samples were equilibrated at 70 °C for 5 min before two consecutive thermal cycles (70 °C – 10 °C – 70 °C) were performed at a cooling/heating rate of 2.0 °C min^–1^.

### Oscillatory rheology

The measurements were performed using an Anton Paar MCR502 rheometer equipped with a Peltier temperature controller, cone-and-plate geometry (a truncated 50 mm 2° stainless steel cone) and TruGap functionality for online monitoring of the geometry gap. The storage (*G*′) and loss (*G*′′) moduli were measured as a function of temperature at a fixed strain amplitude of 1.0% and an angular frequency of 10 rad s^–1^. Thermal cycles between 20 °C and 50 °C were performed at 1.0 °C intervals with an equilibration time of 5 min being allowed prior to each measurement.

### Small-angle and wide-angle X-ray scattering

SAXS and WAXS data were collected simultaneously using a laboratory SAXS/WAXS instrument (Xeuss 2.0, Xenocs, France) equipped with a liquid gallium MetalJet X-ray source (Excillum, Sweden, wavelength *λ* = 0.134 nm), two sets of motorized scatterless slits for beam collimation, a Dectris Pilatus 1M pixel SAXS detector (sample-to-detector distance 1.889 m) and a Dectris Pilatus 100K pixel WAXS detector (sample-to-detector distance 0.178 m, tilted 36° relative to the incident X-ray beam). SAXS and WAXS patterns were recorded over a *q* range of 0.06 nm^–1^ < *q* < 4.0 nm^–1^ and 10.5 nm^–1^ < *q* < 25 nm^–1^ (12.9° < 2*θ* < 31.0°), respectively, where *q* = (4πsin *θ*)/*λ* is the length of the scattering vector and *θ* is one-half of the scattering angle. Glass capillaries of 2 mm diameter were used as a sample holder and the temperature was controlled using a heating/cooling capillary holding stage (Linkam Scientific Instruments Ltd., Tadworth, UK), with 5 min equilibration being allowed prior to data collection over 30 min (for 1.0% w/w dispersions) or 5 min (for 20% w/w dispersions). Data were reduced (normalization and integration) using the Foxtrot software package supplied with the instrument and further analyzed (background subtraction and data modelling) using Irena SAS macros^79^ for Igor Pro.

## Results and discussion

### Synthesis of PBeMA_37_–PBzMA_*x*_ diblock copolymer spheres

**Fig. 1:**
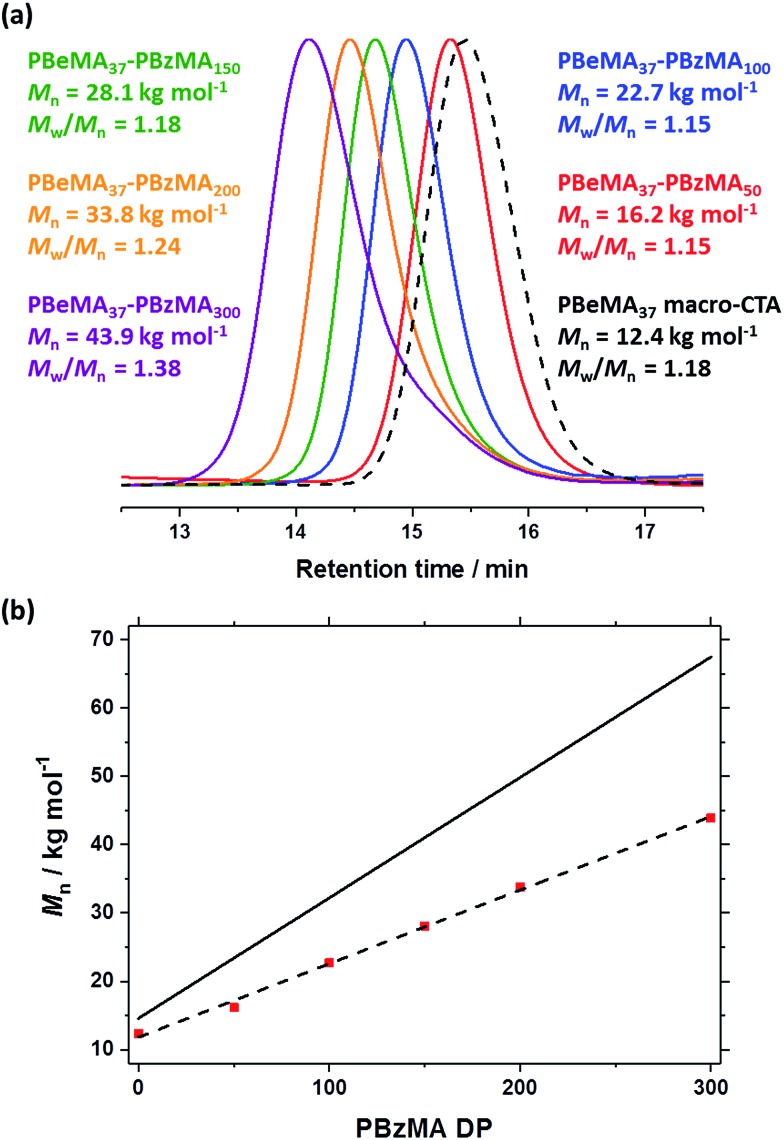
(a) Normalized THF gel permeation chromatograms [against poly(methyl methacrylate) standards] obtained for the series of PBeMA_37_–PBzMA_*x*_ diblock copolymers synthesized *via* RAFT dispersion polymerization of BzMA in mineral oil at 90 °C and 20% w/w solids. The precursor PBeMA_37_ macro-CTA (prepared in toluene at 70 °C and 50% w/w solids) is shown as a reference (black dashed curve). (b) *M*_n_*vs.* PBzMA DP plot for the same PBhMA_37_–PBzMA_*x*_ series, where the *y*-intercept represents the PBhMA_37_ macro-CTA. The solid line represents the theoretical evolution of *M*_n_ with PBzMA DP.

The near-monodisperse PBeMA_37_ macro-CTA (*M*_w_/*M*_n_ = 1.18) obtained from the RAFT solution polymerization of BeMA in toluene at 70 °C was utilized for the RAFT dispersion polymerization of BzMA in mineral oil at 90 °C to produce a range of PBeMA_37_–PBzMA_*x*_ diblock copolymer spheres (see Table 1). High (>99%) BzMA conversions were achieved in all cases, which is consistent with previously reported PISA formulations utilizing PBzMA core-forming blocks.^41^,^48^,^49^,^74^–^77^ Reasonably narrow molecular weight distributions (*M*_w_/*M*_n_ ≤ 1.38) were obtained when targeting PBzMA DPs up to 300. A clear shift in molecular weight was observed for these diblock copolymers relative to the corresponding PBeMA_37_ macro-CTA (see Fig. 1a). Moreover, a linear evolution of *M*_n_ with target PBzMA DP was indicated by THF GPC analysis (see Table 1 and Fig. 1b). Interestingly, all dispersions formed turbid, free-standing gels/pastes immediately after cooling from 90 °C to 20 °C (see Fig. S2a†), whereas free-flowing fluids of varying turbidity were observed on reheating to 50 °C (see Fig. S2b†). This thermal transition proved to be fully reversible.

**Table 1:** Summary of targeted copolymer compositions, BzMA conversions (% BzMA) as judged by ^1^H NMR spectroscopy, GPC and DLS data (particle diameter and polydispersity index, PDI) obtained for a series of PBeMA_37_–PBzMA_*x*_ diblock copolymers prepared by RAFT dispersion polymerization of BzMA in mineral oil. Synthesis conditions: 90 °C, [PBeMA_37_ macro-CTA]/[T21s] molar ratio = 5.0, 20% w/w solids. Relevant data for the PBeMA_37_ macro-CTA are also shown for reference

Target composition	% BzMA	THF GPC (*vs.* PMMA)		DLS at 50 °C	
		*M*_n_/g mol^–1^	*M*_w_/*M*_n_	Particle diameter nm^–1^	Polydispersity index
PBeMA_37_	—	12 400	1.18	—	—
PBeMA_37_–PBzMA_50_	>99	16 200	1.15	21	0.08
PBeMA_37_–PBzMA_100_	>99	22 700	1.15	32	0.01
PBeMA_37_–PBzMA_150_	>99	28 100	1.18	37	0.02
PBeMA_37_–PBzMA_200_	>99	33 800	1.24	55	0.01
PBeMA_37_–PBzMA_300_	>99	43 900	1.38	67	0.01

**Fig. 2:**
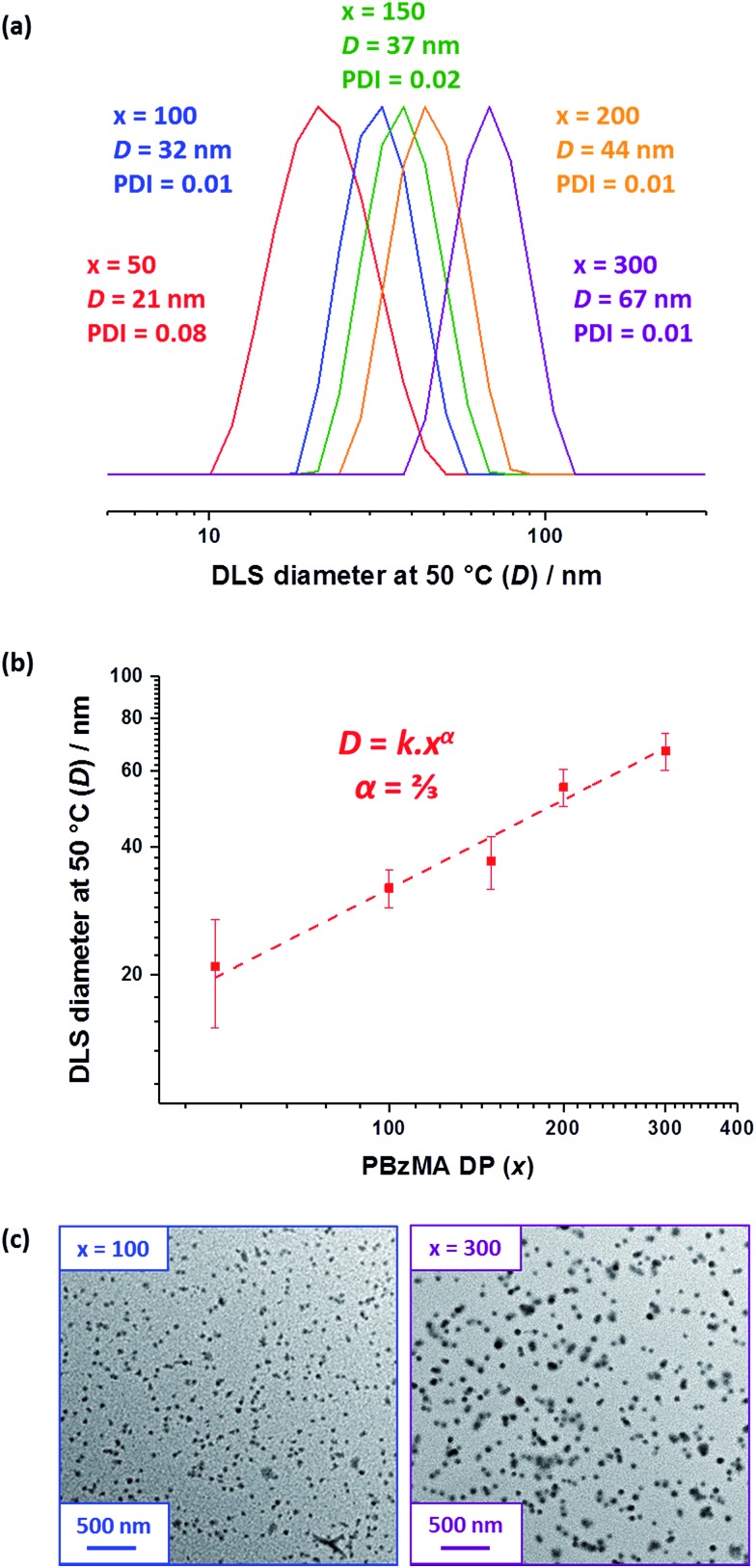
(a) DLS particle size distributions recorded for 0.10% w/w dispersions of PBeMA_37_–PBzMA_*x*_ diblock copolymer spheres in *n*-dodecane at 50 °C. (b) The apparent hydrodynamic diameter (*D*) determined by DLS at 50 °C *vs.* PBzMA DP (*x*) plot indicates an *α* scaling exponent of 2/3. (c) TEM images for selected PBeMA_37_–PBzMA_*x*_ spheres obtained after drying 0.10% w/w dispersions in *n*-dodecane. See Fig. S3† for additional higher magnification TEM images.

**Fig. 3:**
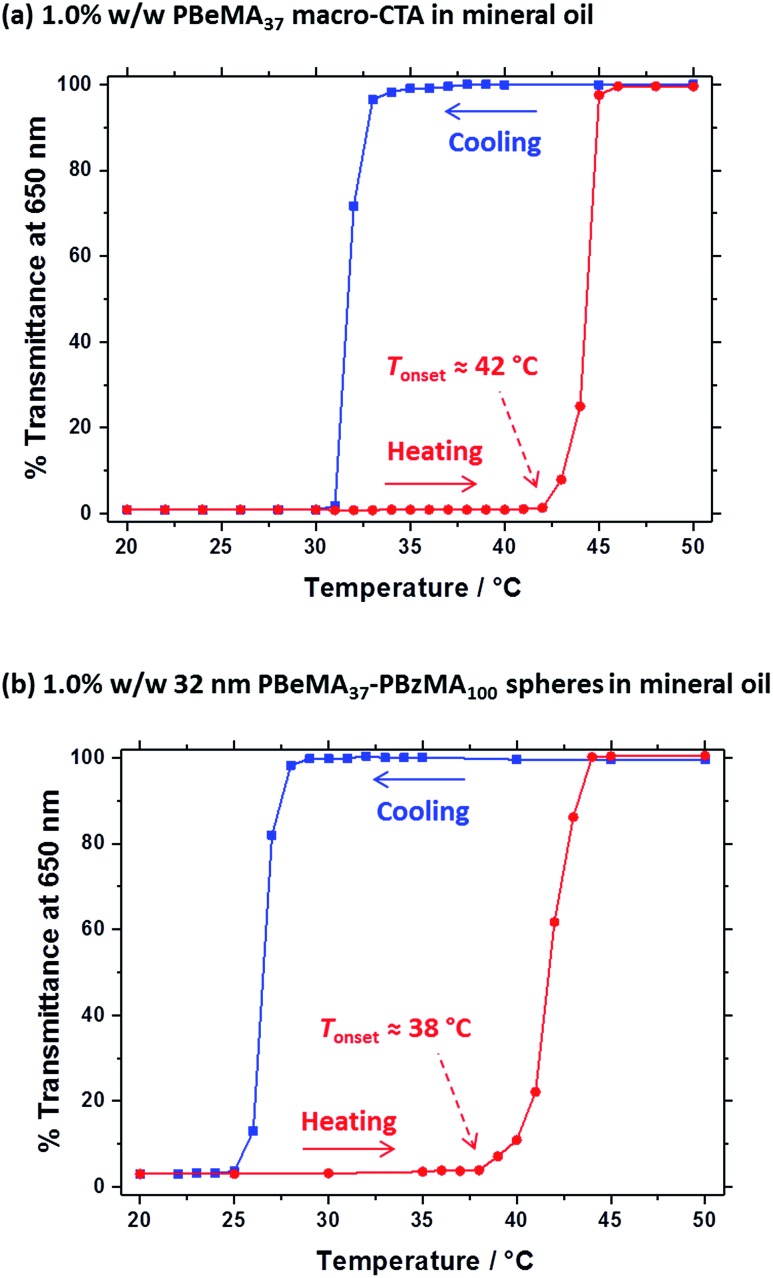
% Transmittance *vs.* temperature plots for (a) a 1.0% w/w solution of the PBeMA_37_ macro-CTA and (b) a 1.0% w/w dispersion of 32 nm PBeMA_37_–PBzMA_100_ spheres in mineral oil. The optical transmittance was monitored at a fixed wavelength of 650 nm on cooling from 50 °C to 20 °C (blue squares) and on heating from 20 °C to 50 °C (red circles), with 5 min being allowed for equilibration at each temperature. Solid lines are shown as a guide to the eye.

DLS studies were performed at 50 °C to determine the mean particle diameter for 0.10% w/w PBeMA_37_–PBzMA_*x*_ dispersions in *n*-dodecane (see Table 1, and Fig. 3a and b). All dispersions exhibited narrow size distributions (PDI < 0.10) and a monotonic increase in apparent hydrodynamic particle diameter (*D*) was observed when targeting a higher PBzMA DP (*x*), where *D* = *kx*^*α*^ (see Fig. 2b). The scaling exponent (*α*) of 2/3 indicates that the PBeMA_37_–PBzMA_*x*_ diblock copolymer chains lie within the strong segregation regime.^80^ This scaling relationship enables reproducible targeting of spheres with a predetermined diameter.^75^,^76^ TEM images confirm that well-defined spherical morphologies were obtained in all cases (see Fig. 2c). It is emphasized that well-defined PBeMA_37_–PBzMA_*x*_ spheres of controllable diameter can be prepared despite the significant loss of control over the molecular weight distribution that occurs when targeting higher PBzMA DPs.

### Thermoreversible crystallization-driven aggregation of PBeMA_37_–PBzMA_*x*_ diblock copolymer spheres

**Fig. 4:**
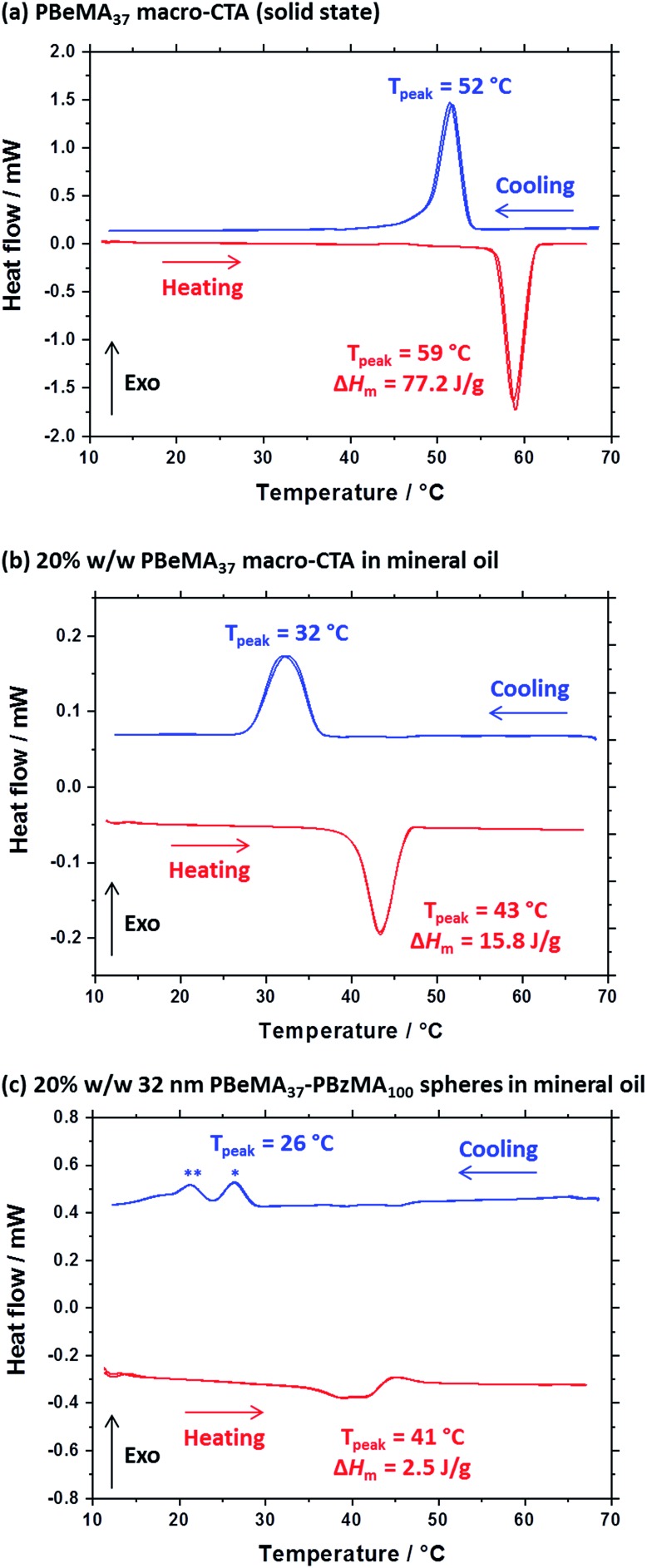
Differential scanning calorimetry (DSC) experiments conducted at a cooling/heating rate of 2 °C min^–1^ for (a) PBeMA_37_ macro-CTA in the solid state, (b) a 20% w/w solution of PBeMA_37_ in mineral oil and (c) a 20% w/w dispersion of 32 nm PBeMA_37_–PBzMA_100_ spheres in mineral oil. Two thermal cycles beginning at the maximum temperature were performed and are presented on the plot. Blue and red data represent cooling and heating ramps, respectively. * Indicates the behenyl side-chain crystallization within individual (isolated) nanoparticles. ** Indicates the secondary crystallization between PBeMA_37_–PBzMA_100_ nanoparticles.

A series of experiments were conducted to determine precisely how the PBeMA_37_ stabilizer block dictates the colloidal stability of PBeMA_37_–PBzMA_*x*_ spheres in mineral oil. Firstly, turbidimetry measurements were performed on a 1.0% w/w solution of PBeMA_37_ macro-CTA in mineral oil (Fig. 3a) and a 1.0% w/w dispersion of 32 nm PBeMA_37_–PBzMA_100_ spheres in mineral oil (Fig. 3b). At 50 °C, approximately 100% transmittance was observed in both cases. This indicates that the PBeMA chains are fully soluble and thus the sterically-stabilized spheres should be well dispersed and non-interacting under the same conditions.^81^ On cooling to below 32 °C, the PBeMA_37_ chains precipitate from mineral oil and the initially clear solution becomes turbid (∼0% transmittance). As anticipated for a first-order phase transition, thermal hysteresis is observed on heating; the critical temperature for redissolution is approximately 45 °C (see Fig. 3a). Similar behavior is also observed for a 1.0% w/w dispersion of 32 nm PBeMA_37_–PBzMA_100_ spheres. However, in this case cooling from 50 °C leads to nanoparticle flocculation rather than homopolymer precipitation. Moreover, the critical temperature for this phase transition is approximately 27 °C (see Fig. 3b). In principle, this difference may be partly attributable to the lower concentration of PBeMA_37_ stabilizer chains in this dispersion (∼0.45% w/w) compared to that of the PBeMA_37_ solution (1.0% w/w). Alternatively, crystallization may be suppressed because one end of each crystallizing PBeMA_37_ stabilizer chain is bound to an interface (the PBzMA nanoparticle cores). The melting temperature required for reconstitution of a colloidally stable dispersion on heating is approximately 42 °C (see Fig. 3b), which is comparable to the critical temperature required for redissolution of the free PBeMA_37_ stabilizer chains (see Fig. 3a). We hypothesize that the strong hysteresis observed in the present study arises primarily because flocculation is driven by crystallization of the alkyl side chains on the PBeMA stabilizer, which involves a first-order phase transition as opposed to a second-order phase transition (*i.e.* liquid–liquid phase separation). Similar local ordering has been previously reported for closely-related poly(*n*-alkyl methacrylates) such as poly(stearyl methacrylate): DSC studies indicate that side-chain crystallization occurs in the solid state for both this homopolymer and also for several poly(stearyl methacrylate)-based diblock copolymers.^82^,^83^ To test our hypothesis of crystallization-driven flocculation, we performed DSC experiments on (i) the PBeMA_37_ precursor in the solid state (Fig. 4a), (ii) a 20% w/w PBeMA_37_ solution in mineral oil (Fig. 4b) and (iii) a 20% w/w dispersion of 32 nm PBeMA_37_–PBzMA_100_ spheres in mineral oil (Fig. 4c). For the PBeMA_37_ homopolymer in the solid state, a melting enthalpy (Δ*H*_m_) of 77.2 J g^–1^ corresponding to the crystalline behenyl side-chains indicates a mean degree of crystallinity of ∼31% relative to the theoretical value reported for the polyethylene unit cell.^84^ The Δ*H*_m_ for PBeMA_37_ in a 20% w/w mineral oil solution is reduced approximately five-fold to 15.8 J g^–1^, simply owing to dilution. The same transitions can be observed in both cases, although the exotherm/endotherm becomes much weaker and the transition temperatures are suppressed for lower concentrations of PBeMA_37_ chains, as expected. These transitions can be attributed to the crystallization/melting of the behenyl side-chains within the PBeMA_37_ block. Interestingly, multiple peaks were observed in the DSC traces recorded for a 20% w/w dispersion of 32 nm PBeMA_37_–PBzMA_100_ spheres in mineral oil (see Fig. 4c). These features indicate subtly different crystallization events. More specifically, behenyl side-chains within individual (isolated) nanoparticles crystallize first (see peak at ∼26 °C in Fig. 4c), before then acting as nucleation sites for neighboring nanoparticles (see peak at ∼21 °C in Fig. 4c), thus leading to the formation of colloidal aggregates of strongly-interacting PBeMA_37_–PBzMA_100_ nanoparticles.

**Fig. 5:**
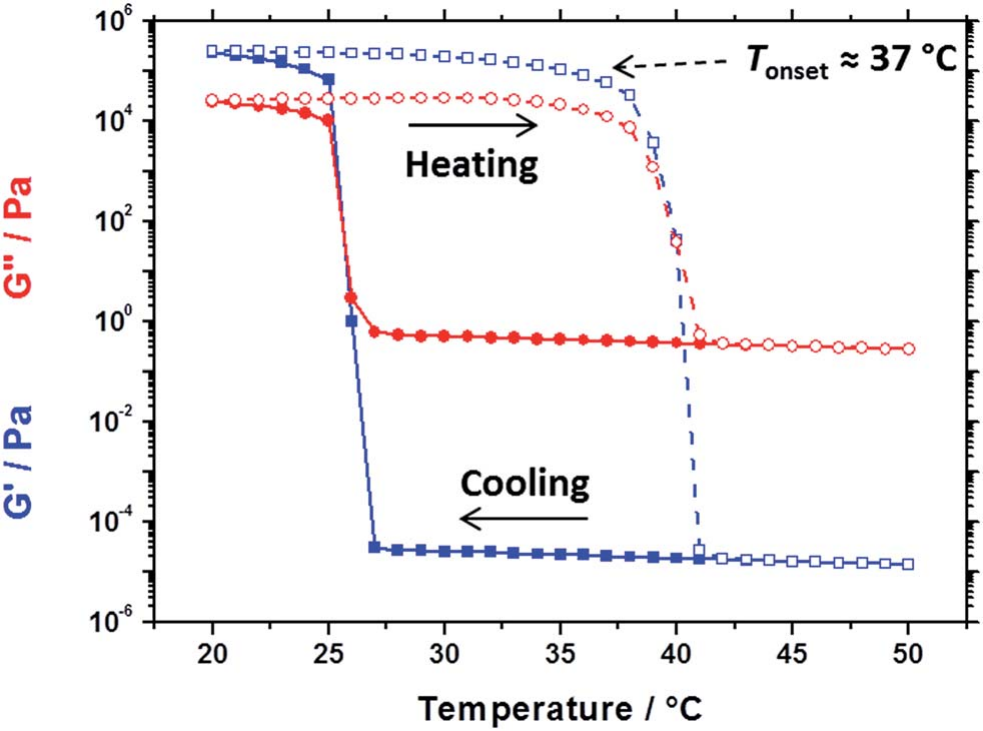
Temperature dependence of the storage modulus (*G*′, blue squares) and loss modulus (*G*′′, red circles) for a 20% w/w dispersion of 32 nm PBeMA_37_–PBzMA_100_ spherical nanoparticles in mineral oil on cooling from 50 °C to 20 °C (filled symbols) and on heating from 20 °C to 50 °C (open symbols). Data were recorded at 1.0% strain amplitude using an angular frequency of 10 rad s^–1^, with 5 min being allowed for thermal equilibration between each measurement.

Oscillatory rheology experiments performed on the same 20% w/w dispersion of 32 nm PBeMA_37_–PBzMA_100_ spheres in mineral oil (see Fig. 5) are consistent with the transition temperatures determined by DSC (26 °C on cooling and 41 °C on heating, Fig. 4c). On cooling from 50 °C to 27 °C, the 20% w/w dispersion exhibited fluid-like properties, with the loss modulus (*G*′′) being much larger than the storage modulus (*G*′). The liquid-to-solid transition is defined by the crossover in the *G*′ and *G*′′ curves at 25 °C, which occurs on further cooling. Hysteresis is observed as the dispersion is heated from 20 °C to 50 °C: its solid-like properties are initially retained (*G*′ > *G*′′) before reverting to its original fluid-like state (*G*′′ ≫ *G*′) at 41 °C. Despite this hysteresis, a fully thermoreversible transition is observed. These results also support those depicted in the optical digital images shown in Fig. S2:† free-flowing dispersions are obtained at 50 °C, while solid paste-like dispersions are formed at 20 °C. Moreover, the rheology and DSC studies performed on the 20% w/w dispersion of PBeMA_37_–PBzMA_100_ nanoparticles correlate well with the optical transmittance data recorded for the corresponding 1.0% w/w dispersion (Fig. 3b).

### X-ray scattering studies

**Fig. 6:**
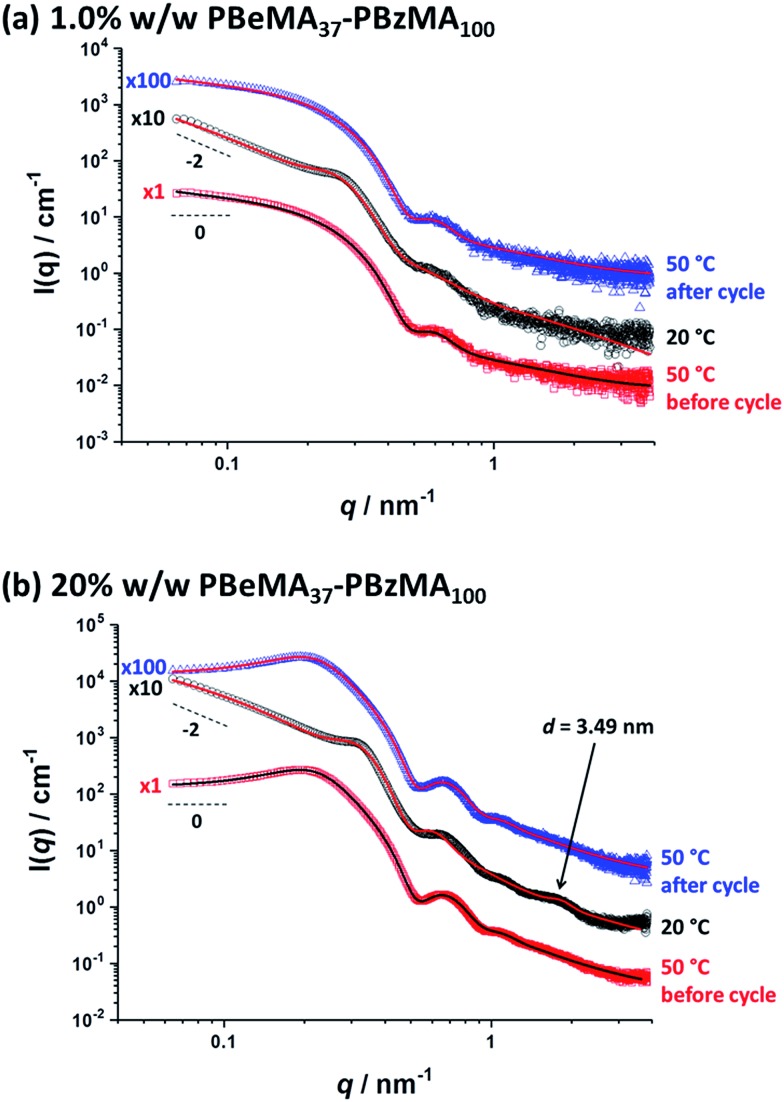
Small-angle X-ray scattering (SAXS) patterns recorded for (a) a 1.0% w/w dispersion and (b) a 20% w/w dispersion of PBeMA_37_–PBzMA_100_ spheres in mineral oil at 50 °C before the thermal cycle (red squares), 20 °C after cooling (black circles) and 50 °C after reheating (blue triangles). Solid lines indicate relevant data fits to an established spherical micelle model (see ESI†),^85^ with a sticky hard-sphere^87^ or a Percus–Yevick hard-sphere^88^ structure factor being incorporated where appropriate. Patterns are offset by an arbitrary factor (indicated on the left side of the patterns) for clarity.

Variable-temperature SAXS studies were conducted to further probe the aggregation behavior of the PBeMA_37_–PBzMA_100_ nanoparticles (see Fig. 6). Initially, data were collected for a dilute 1.0% w/w dispersion; inter-particle interactions are negligible under such conditions so the particle form factor could be estimated. At 50 °C (see Fig. 6a, red squares), the zero gradient observed at low *q* in the *I*(*q*) *vs. q* plot is consistent with non-interacting spherical particles. Moreover, this pattern can be well-fitted using a spherical micelle model (see SAXS model section in the ESI†),^85^ which indicates a spherical core radius (*R*_s_) of 8.1 ± 1.0 nm and a radius of gyration (*R*_g_) for the PBeMA_37_ stabilizer chains of 1.49 nm (see Fig. 6a and Table 2). The micelle core diameter (*D*_c_ = 2*R*_s_) of 16.2 ± 2.0 nm correlates well with the TEM image shown in Fig. S3† which indicates a mean core diameter of 16.2 ± 1.8 nm (estimated from analysis of 50 nanoparticles). The corresponding volume-average diameter *D*_v_ is 22.1 ± 3.7 nm (where *D*_v_ = 2*R*_s_ + 4*R*_g_), which is somewhat smaller than the hydrodynamic *z*-average diameter of 32 nm reported by DLS (see Table 1). In addition, SAXS analysis indicates that the volume fraction of solvent within the nanoparticle cores (*x*_sol_) is essentially zero. Similar results have been recently reported for other diblock copolymer nanoparticles comprising PBzMA core-forming blocks in both *n*-dodecane^48^ and mineral oil.^76^

**Table 2:** Summary of parameters after fitting SAXS patterns obtained for 1.0% w/w and 20% w/w dispersions of PBeMA_37_–PBzMA_100_ nanoparticles in mineral oil at 50 °C and 20 °C using a spherical micelle model (see ESI),^85^ incorporating a sticky hard-sphere^87^ or a Percus–Yevick hard-sphere^88^ structure factor, where appropriate. *φ* is the volume fraction of copolymers obtained from the fitting, *R*_s_ is the spherical micelle core radius, *R*_g_ is the radius of gyration of the PBeMA stabilizer block and the volume-average nanoparticle diameter, *D*_v_, is subsequently calculated using *D*_v_ = 2*R*_s_ + 4*R*_g_. *N*_s_ is the average number of copolymer chains per micelle, equal to 
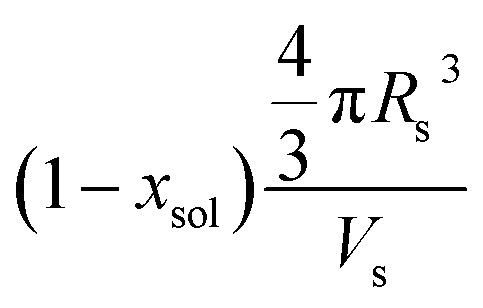
, where *x*_sol_ is the volume fraction of solvent within the core domain (equal to 0 in all cases), and *V*_s_ is the molecular volume of one core-forming PBzMA chain 
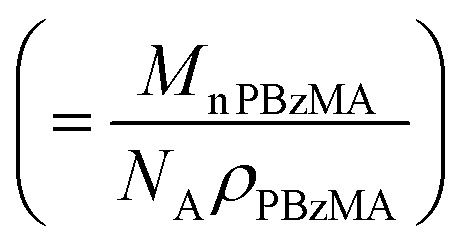
. *S*_agg_ is the number of copolymer chains per unit surface area at the micelle core, equal to 
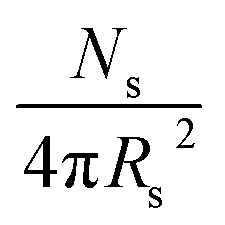
, and *d*_int_ is the average distance between neighboring chains at the core-corona interface, equal to 
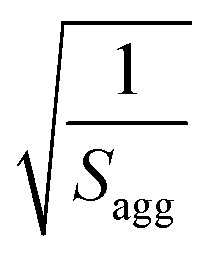
. *D*_SHS_ is the mean interaction distance and *f*_SHS_ is the effective volume fraction of interacting spheres calculated by fitting the sticky hard-sphere model with a stickiness parameter (*τ*) of 0.10, while *D*_PY_ is the mean interaction distance and *f*_PY_ is the effective volume fraction of interacting spheres returned from fitting to the hard-sphere structure factor. The fractal dimension, *p*, describes the upturn in scattering intensity at low *q*

Copolymer concentration/% w/w	*T*/°C	*φ*	*R*_s_/nm	*R*_g_/nm	*D*_v_/nm	*N*_s_	*S*_agg_/nm^–2^	*d*_int_/nm	*D*_SHS_/nm	*f*_SHS_	*D*_PY_/nm	*f*_PY_	*p*
1.0	50	0.00824	8.1 ± 1.0	1.49	22.1 ± 3.7	86 ± 18	0.11 ± 0.03	3.1 ± 0.8	—	—	—	—	—
1.0	20	0.00772	8.1 ± 1.6	0.95*	19.9 ± 5.4	86 ± 29	0.11 ± 0.05	3.1 ± 1.3	27.9	0.194	—	—	1.67
20	50	0.135	7.7 ± 0.7	2.45	25.1 ± 3.0	74 ± 11	0.10 ± 0.02	3.2 ± 0.6	—	—	52.1	0.184	—
20	20	0.105	7.7 ± 1.1	2.04*	23.6 ± 4.9	76 ± 19	0.10 ± 0.03	3.2 ± 1.0	22.9	0.374	—	—	1.67

On cooling to 20 °C, a local maximum in scattering intensity was observed at *q* ∼ 0.26 nm^–1^ (see Fig. 6a, black circles). Moreover, there is an upturn in scattering intensity at low *q*, suggesting the formation of scattering objects that are significantly larger than the individual nanoparticles. These observations indicate that the nanoparticles form large aggregates on cooling even at a relatively low copolymer concentration (1.0% w/w). Furthermore, the slope of the scattered X-ray intensity at low *q* is approximately –2 (see Fig. 6), which is consistent with the formation of mass fractals^86^ by a network of interconnected spherical nanoparticles. This is consistent with the solid-like properties of a 20% w/w PBeMA_37_–PBzMA_100_ dispersion at 20 °C indicated by rheology (see Fig. 5), the digital images of the dispersions (see Fig. S2†), and the transmission electron micrographs obtained for a dilute dispersion at 20 °C (see Fig. S3c†). Dividing the scattering pattern obtained at 20 °C with that recorded at 50 °C reveals the structure factor [see *S*(*q*) curve in Fig. S4a†], which is attributed to partial crystallization of the PBeMA_37_ coronal blocks between neighboring nanoparticles. Fitting the latter data to a sticky hard-sphere (SHS) model^87^ indicated a mean centre-to-centre interaction distance (*D*_SHS_) of 26.7 nm and an interacting sphere volume fraction (*f*_SHS_) of 0.078 (see Fig. S4a†). This approach does not provide a satisfactory fit, but this is not unexpected given that the two data sets used in this analysis were collected at different temperatures. Thus the volumetric contraction of the nanoparticles that occurs on cooling introduces a systematic error. Using the fitting parameters obtained for the colloidally stable 1.0% w/w dispersion at 50 °C and these *D*_SHS_ and *f*_SHS_ values as a starting point, the SAXS pattern obtained for the same flocculated 1.0% w/w dispersion at 20 °C was fitted. The primary nanoparticle core dimensions remain more or less unaffected, with an *R*_s_ of 8.1 ± 1.6 nm being observed at this lower temperature (see Fig. 6a and Table 2). However, the radius of gyration, *R*_g_, returned for the PBeMA_37_ stabilizer block when fitting the SAXS pattern recorded at 20 °C is lower than that indicated by the corresponding 50 °C pattern. In reality, these partially crystalline PBeMA chains are not expected to exhibit Gaussian behavior at 20 °C since their mobility is confined by interactions with neighboring PBeMA chains within the crystal. In addition, such crystallization should lead to an increase in mass density (and hence scattering length density) compared to the non-crystalline stabilizer chains at 50 °C. However, this rather subtle effect is not included in the spherical micelle model. This fitting strategy returned a *D*_SHS_ of 27.9 nm and an *f*_SHS_ of 0.194 (see Table 2). Importantly, the scattering pattern obtained on reheating to 50 °C (see Fig. S4a,† blue triangles) overlaps almost perfectly with that observed for the original spheres at 50 °C (see Fig. S4a,† red squares), which confirms the fully reversible nature of this thermal transition.

Further SAXS analysis was conducted to confirm that the temperature-dependent behavior observed for the 1.0% w/w copolymer dispersion in mineral oil is consistent with that found for the 20% w/w dispersion of PBeMA_37_–PBzMA_100_ spheres. Firstly, data were collected at 50 °C (see Fig. 6b, red squares) and fitted using the same spherical micelle model^85^ (see Table 2). The sticky hard-sphere model used at 20 °C cannot be employed to represent the inter-particle interactions at 50 °C, since this is above the melting temperature of the partially crystalline PBeMA stabilizer chains, hence an amorphous free-flowing dispersion is obtained. Thus, a simple hard-sphere interaction based on the Percus–Yevick (PY) approximation suffices under such conditions.^88^ The numerical values for *R*_s_ (7.7 ± 0.7 nm) and *D*_v_ (25.1 ± 3.0 nm) returned from this fit are in reasonably good agreement with those obtained for the 1.0% w/w dispersion (see Table 2). The PY approximation indicates that a significant fraction of these PBeMA_37_–PBzMA_100_ spheres (*f*_PY_ = 0.184) are strongly interacting, with a mean centre-to-centre interaction distance (*D*_PY_) of 52.1 nm. Moreover, the latter parameter is larger than *D*_v_, which indicates that sphere–sphere interactions arise purely from their proximity in space. On cooling to 20 °C (see Fig. 6b, black circles), *R*_s_ remained relatively unchanged (7.7 ± 1.1 nm), which corresponds to a *D*_v_ of 23.6 ± 4.9 nm. On cooling from 50 °C to 20 °C, the mean interaction distance is reduced from *D*_PY_ = 52.1 nm to *D*_SHS_ = 22.9 nm. The latter value is less than *D*_v_ at 20 °C, which provides compelling (albeit indirect) evidence that the interparticle interactions involve crystallization between PBeMA stabilizer chains located on neighboring spherical nanoparticles. The upturn in scattering intensity at low *q* is similar to that observed for the 1.0% w/w dispersion and again indicates the formation of loose mass fractals.^86^

**Fig. 7:**
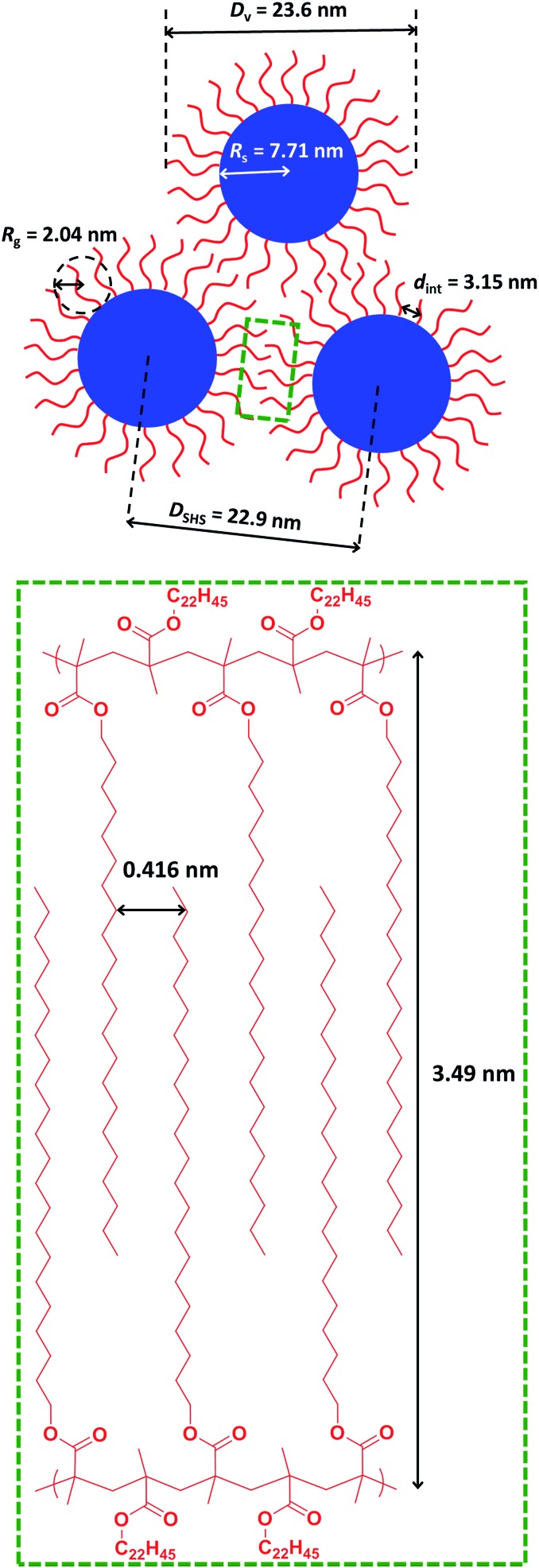
Schematic representation of the characteristic length scales for the structural parameters associated with the formation of loose mass fractal aggregates *via* crystallization-driven self-assembly (CDSA) of a 20% w/w dispersion of PBeMA_37_–PBzMA_100_ nanoparticles in mineral oil at 20 °C. The mean *d*-spacing of 0.416 nm between co-crystallizing behenyl side-chains is calculated from WAXS analysis, whereas all other parameters are derived from SAXS analysis (see main text for further details).

Direct evidence for behenyl side-chain crystallization between neighboring PBeMA chains is provided by the subtle feature at *q* = 1.80 nm^–1^ in the SAXS pattern recorded for a 20% w/w dispersion at 20 °C (Fig. 6b), which indicates a mean *d*-spacing of 3.49 nm (*d* = 2π/*q*). This characteristic length scale corresponds to a periodic lamellar structure formed by co-crystallizing methacrylic backbones and is similar to that reported for poly(stearyl methacrylate).^82^ Assuming every monomer repeat unit comprises two C–C bonds in an all-trans conformation, the theoretical maximum length of one fully-stretched behenyl side-chain is 2.81 nm. Hence the distance between non-interacting side-chains should be greater than 5.62 nm. Thus, a mean spacing of 3.49 nm implies significant interdigitation between PBeMA stabilizer chains on neighboring nanoparticles (see Fig. 7). This interaction distance is physically reasonable when compared to the mean distance between adjacent diblock copolymer chains located at the nanoparticle surface (*d*_int_), which is calculated to be 3.2 nm for this 20% w/w dispersion at 20 °C (see Table 2).

WAXS patterns (recorded simultaneously with the SAXS data) were used to examine crystallization between the behenyl side-chains at shorter length scales. Comparing WAXS data recorded for the 20% w/w dispersion of PBeMA_37_–PBzMA_100_ nanoparticles at 20 °C to that obtained for mineral oil alone revealed a discernible broad peak at *q* = 15.1 nm^–1^ for the former system (see Fig. S5†). This corresponds to a mean *d*-spacing of 0.416 nm (see Fig. 7), which is in good agreement with the 100 reflection reported for the hexagonal rotator phase of *n*-alkanes,^89^ and also with that reported for the closely-related poly(stearyl methacrylate).^82^ A schematic representation of the characteristic length scales associated with the formation of loose mass fractal aggregates *via* crystallization-driven self-assembly of sterically-stabilized nanoparticles is shown in Fig. 7.

It is proposed that the onset of crystallization involves homogeneous nucleation within *isolated* PBeMA_37_–PBzMA_100_ nanoparticles, hence substantial undercooling is required.^90^ These crystalline domains decorate the nanoparticles, hence any contact with neighboring nanoparticles *via* Brownian motion initiates secondary nucleation, which is rapid compared to nanoparticle diffusion. Thus nanoparticles impinging on such crystalline domains rapidly crystallize and stick, quickly leading to the formation of loose fractal aggregates *via* reaction-limited aggregation.^91^ This mechanism is consistent with the DSC and rheology measurements, whereby crystallization and gelation are observed under similar conditions for the same 20% w/w dispersion of PBeMA_37_–PBzMA_100_ spheres. Importantly, when this relatively concentrated dispersion is returned to 50 °C, its new scattering pattern overlaps perfectly with that initially obtained at 50 °C (see Fig. S4†), confirming full reversibility for this crystallization-driven thermal transition. It is perhaps worth emphasizing that such CDSA-mediated nanoparticle aggregation behavior distinguishes the current study from the various literature reports of upper critical solution temperature (UCST)-driven aggregation of sterically-stabilized nanoparticles in which the amorphous stabilizer block becomes insoluble on lowering the temperature.^92^

## Conclusions

In summary, a series of PBeMA_37_–PBzMA_*x*_ spherical nanoparticles are prepared *via* RAFT dispersion polymerization of benzyl methacrylate at 90 °C in mineral oil at 20% w/w solids. On cooling to 20 °C, turbid pastes are formed for all PBeMA_37_–PBzMA_*x*_ spheres in mineral oil as a result of the crystallization of the insoluble PBeMA stabilizer block. However, heating to 50 °C leads to free-flowing dispersions, with DLS studies performed at this temperature indicating narrow size distributions and a strong correlation between the mean degree of polymerization of the core-forming PBzMA block and the *z*-average nanoparticle diameter. On cooling from 50 °C to 20 °C, turbidimetry studies conducted on a 1.0% w/w solution of the PBeMA_37_ macro-CTA in mineral oil indicated that precipitation occurred at around 32 °C, with redissolution occurring at around 45 °C on reheating. Thus this control experiment demonstrates that the thermosensitive nature of the PBeMA_37_ stabilizer chains determines the colloidal stability of the PBeMA_37_–PBzMA_*x*_ spheres. Oscillatory rheology experiments were performed to study the sol–gel behavior of 20% w/w PBeMA_37_–PBzMA_100_ nanoparticles in mineral oil: a free-flowing fluid of non-interacting spheres was observed at 50 °C, whereas a solid-like paste of strongly-interacting spheres was obtained on cooling to 20 °C. Notably, critical transition temperatures for the same 20% w/w dispersion of PBeMA_37_–PBzMA_100_ spheres in mineral oil determined *via* DSC and rheology were in excellent agreement. SAXS studies confirmed that PBeMA_37_–PBzMA_100_ spheres in mineral oil became strongly interacting and formed loose mass fractals at 20 °C. Importantly, the mean interaction distance for spherical nanoparticles at 20 °C was less than the volume-average diameter of the spheres, thus providing indirect evidence for crystallization between PBeMA chains on neighboring nanoparticles. Furthermore, WAXS studies indicated a crystalline hexagonal rotator phase between PBeMA chains at 20 °C, with a mean *d*-spacing of 4.16 Å between 100 planes formed by co-crystallizing behenyl side-chains. In contrast, well-dispersed nanoparticles are obtained at 50 °C and this thermal transition is fully reversible. However, pronounced hysteresis can be observed under certain conditions owing to the crystallization/melting behavior of the PBeMA stabilizer chains.

## Conflicts of interest

There are no conflicts to declare.

## Supplementary Material

Supplementary information
