# Correction: Thermoreversible crystallization-driven aggregation of diblock copolymer nanoparticles in mineral oil

**DOI:** 10.1039/c8sc90241k

**Published:** 2018-12-20

**Authors:** Matthew J. Derry, Oleksandr O. Mykhaylyk, Anthony J. Ryan, Steven P. Armes

**Affiliations:** a Department of Chemistry , The University of Sheffield , Dainton Building, Brook Hill , Sheffield , South Yorkshire S3 7HF , UK . Email: m.derry@sheffield.ac.uk

## Abstract

Correction for ‘Thermoreversible crystallization-driven aggregation of diblock copolymer nanoparticles in mineral oil’ by Matthew J. Derry *et al.*, *Chem. Sci.*, 2018, **9**, 4071–4082.



## 

The authors regret that in Table 1 the units for particle diameter are incorrect. This should be nm. The correct Table 1 is displayed below.

**Table 1:** Summary of targeted copolymer compositions, BzMA conversions (% BzMA) as judged by ^1^H NMR spectroscopy, GPC and DLS data (particle diameter and polydispersity index, PDI) obtained for a series of PBeMA_37_–PBzMA_*x*_ diblock copolymers prepared by RAFT dispersion polymerization of BzMA in mineral oil. Synthesis conditions: 90 °C, [PBeMA_37_ macro-CTA]/[T21s] molar ratio = 5.0, 20% w/w solids. Relevant data for the PBeMA_37_ macro-CTA are also shown for reference

Target composition	% BzMA	THF GPC (*vs.* PMMA)		DLS at 50 °C	
		*M*_n_/g mol^–1^	*M*_w_/*M*_n_	Particle diameter/nm	Polydispersity index
PBeMA_37_	—	12 400	1.18	—	—
PBeMA_37_–PBzMA_50_	>99	16 200	1.15	21	0.08
PBeMA_37_–PBzMA_100_	>99	22 700	1.15	32	0.01
PBeMA_37_–PBzMA_150_	>99	28 100	1.18	37	0.02
PBeMA_37_–PBzMA_200_	>99	33 800	1.24	55	0.01
PBeMA_37_–PBzMA_300_	>99	43 900	1.38	67	0.01

The Royal Society of Chemistry apologises for these errors and any consequent inconvenience to authors and readers.

