# Supplementary material for: Thermoreversible crystallization-driven aggregation of diblock copolymer nanoparticles in mineral oil
Source: Chem Sci. 2018 Apr 2;9(17):4071–82. doi: 10.1039/c8sc00762d (PMC5944243; doi:10.1039/c8sc00762d)
Supplement: Supplementary file 1 [file SC-009-C8SC00762D-s001.pdf]

Electronic Supplementary Material (ESI) for Chemical Science.
This journal is © The Royal Society of Chemistry 2018

Supporting Information for:

Thermoreversible crystallization-driven aggregation of diblock copolymer nanoparticles in mineral oil

Matthew J. Derry,* Oleksandr O. Mykhaylyk, Anthony J. Ryan and Steven P. Armes*

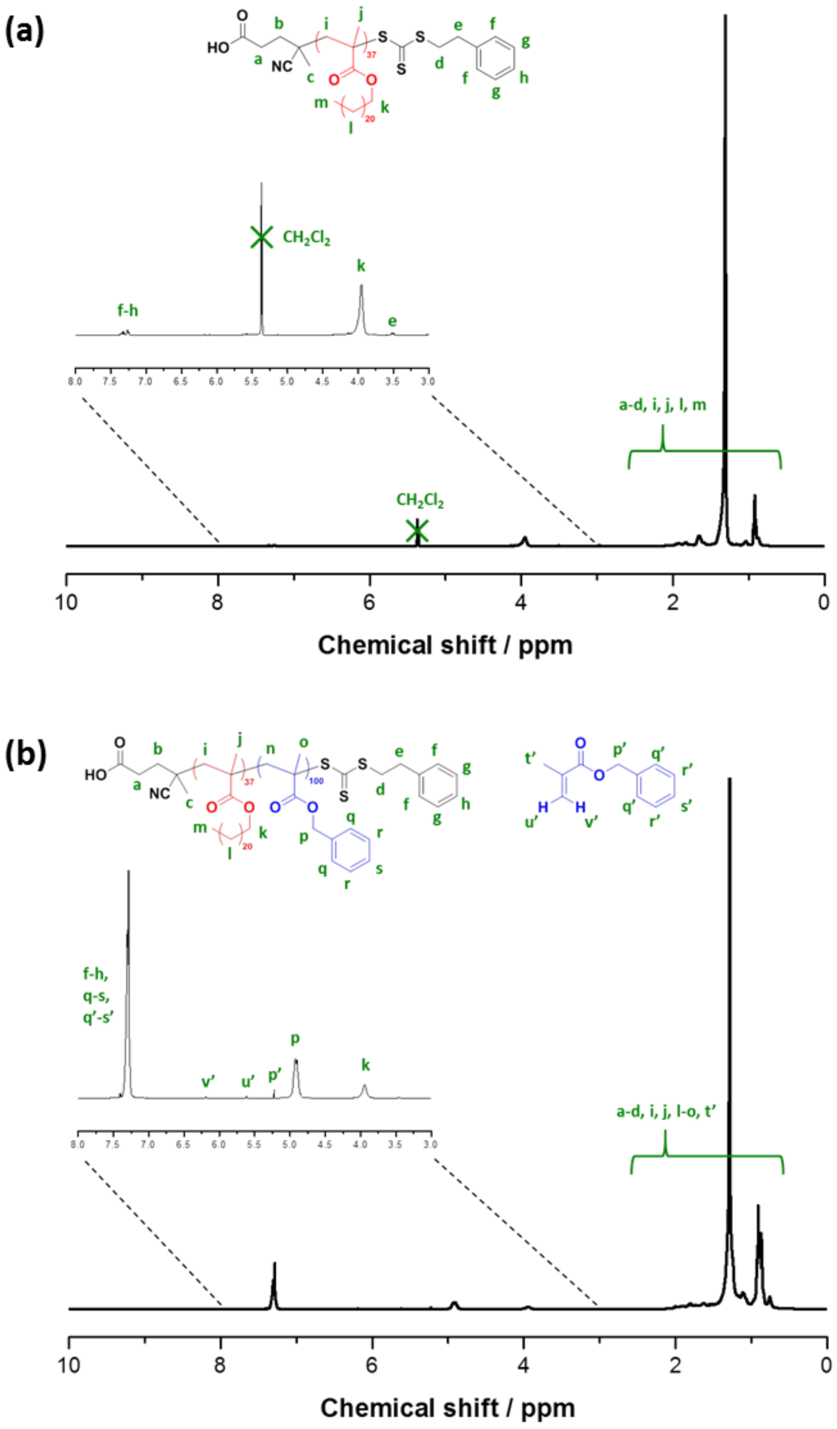

Figure S1. Assigned ^{1}H NMR spectrum for (a) purified PBeMA$_{37}$ macro-CTA in CD_2Cl_2 and (b) PBeMA$_{37}$-PBzMA$_{100}$ in $CDCl_3$.

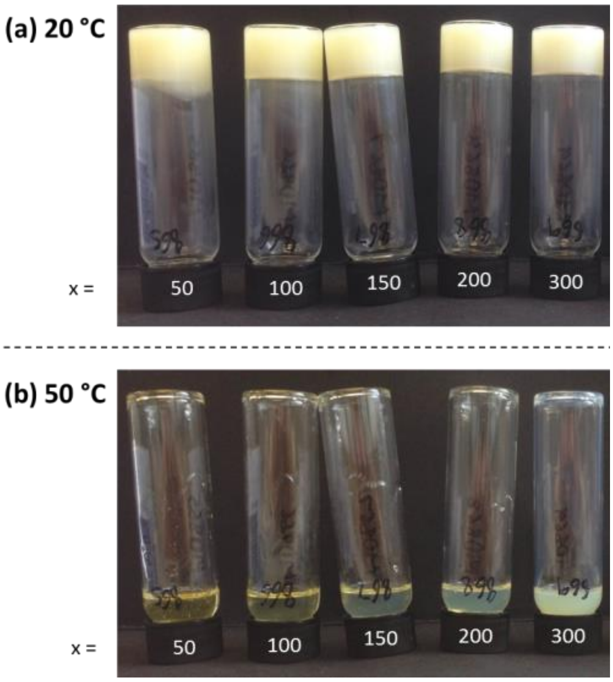

Figure S2. Digital images recorded for a series of 20% w/w dispersions of $PBeMA_{37}$-$PBzMA_x$ spheres prepared in mineral oil at (a) 20 °C and (b) 50 °C.

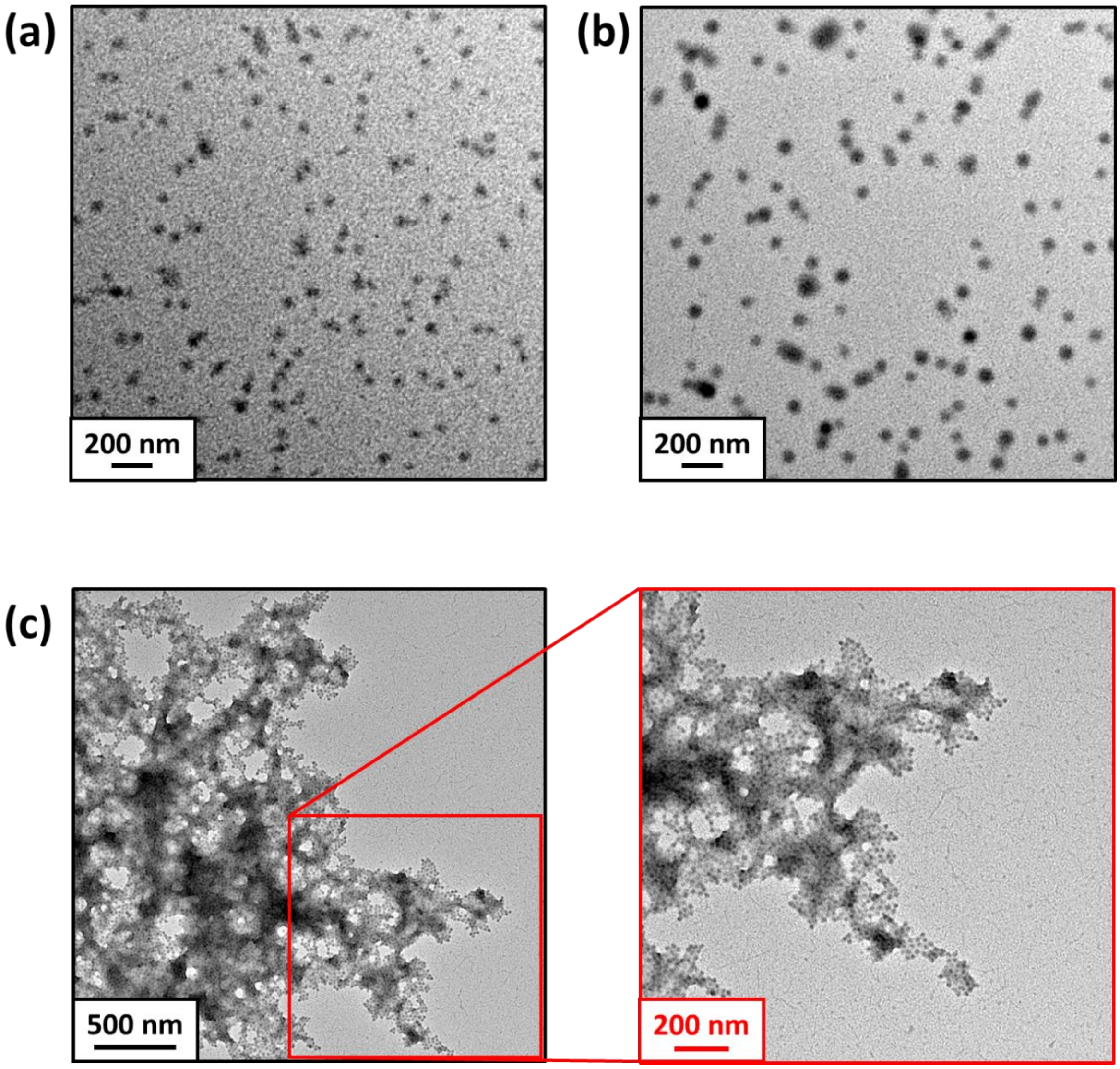

Figure S3. Selected transmission electron micrographs obtained for a 0.10% w/w dispersions of (a) $PBeMA_{37}$-$PBzMA_{100}$ spheres prepared at 50 °C, (b) $PBeMA_{37}$-$PBzMA_{300}$ spheres prepared at 50 °C, and (c) $PBeMA_{37}$-$PBzMA_{100}$ spheres prepared at 20 °C. Image analysis on 50 $PBeMA_{37}$-$PBzMA_{100}$ nanoparticles prepared at 20 °C indicated a mean core diameter of 16.2 ± 1.8 nm, and a sphere-to-sphere distance of 23.5 ± 2.76 nm.

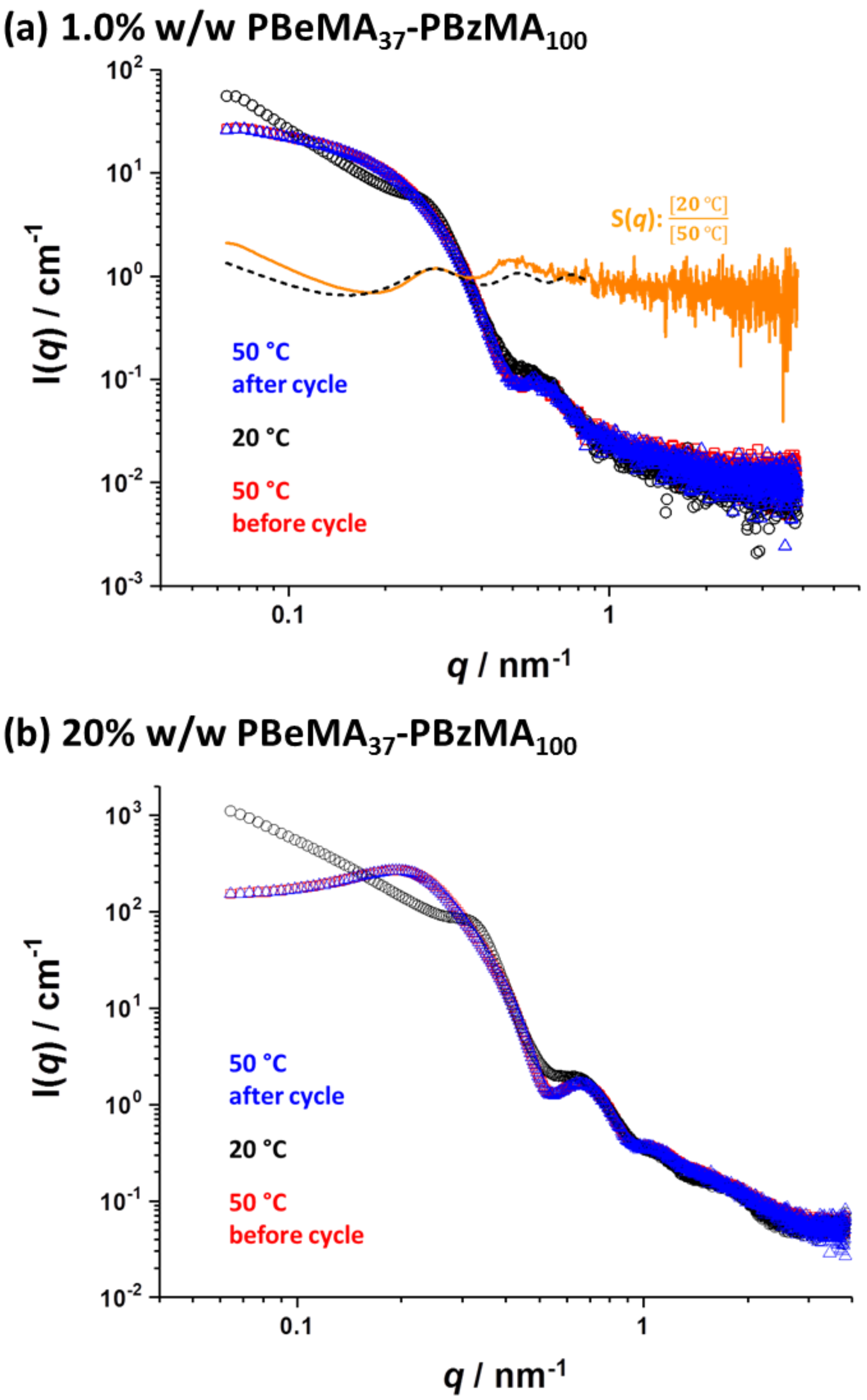

Figure S4. Small-angle X-ray scattering (SAXS) patterns recorded for (a) a 1.0% w/w dispersion and (b) a 20% w/w dispersion of $PBeMA_{37}$-$PBzMA_{100}$ spheres in mineral oil at 50 °C before cooling (red squares), after cooling to 20 °C (black circles) and after reheating to 50 °C (blue triangles). The structure factor [S(q)] observed for a 1.0% w/w dispersion at 20 °C is determined normalizing the pattern obtained at 20 °C relative to that collected at 50 °C for the same dispersion, and is indicated by the orange line in (a), with the fit to the sticky hard-sphere model shown as a black dashed curve.

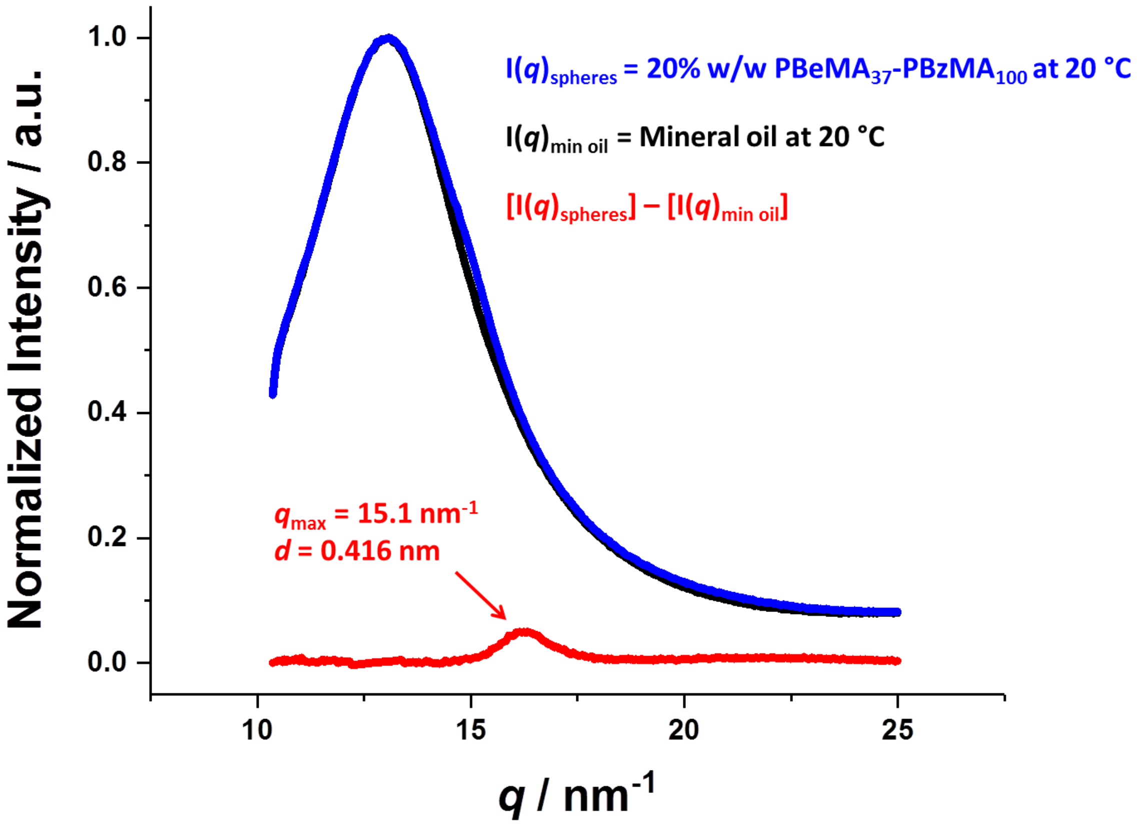

Figure S5. Wide-angle X-ray scattering (WAXS) patterns recorded at 20 °C for a 20% w/w dispersion of $PBeMA_{37}$-$PBzMA_{100}$ spheres in mineral oil (blue data), mineral oil (black data) and the difference between these patterns (red data). The first two experimental patterns were normalized by their maximum intensities.

SAXS models

In general, the X-ray intensity scattered by a dispersion of particles [usually represented by the scattering cross section per unit sample volume, $\frac{d\Sigma}{d\Omega}(q)$] can be expressed as:

$$\frac{d\Sigma}{d\Omega}(q) = NS_{\mathrm{SF}}(q)\int_0^\infty \dots \int_0^\infty F(q, r_{1,} \dots, r_{\mathrm{k}})^2 \Psi(r_{1,} \dots, r_{\mathrm{k}}) dr_1 \dots dr_{\mathrm{k}} \qquad S1$$

where $F(q, r_1, \dots, r_{\mathrm{k}})$ is their form factor, $r_1, \dots, r_{\mathrm{k}}$ is a set of k parameters describing the particle structural morphology, $\Psi(r_{1,} \dots, r_{\mathrm{k}})$ is the distribution function, $S_{\mathrm{SF}}(q)$ is the structure factor and N is the particle number density per unit sample volume expressed as:

$$N = \frac{\varphi}{\int_0^\infty \dots \int_0^\infty V(r_{1,} \dots, r_{\mathrm{k}})\, \Psi(r_{1,} \dots, r_{\mathrm{k}}) dr_1 \dots dr_{\mathrm{k}}} \qquad S2$$

where $V(r_{1,} \dots, r_{\mathrm{k}})$ is volume of the particle and φ is their volume fraction in the dispersion.

The spherical micelle form factor for Equation *S1* can be expressed as:[1]

$$\begin{aligned} F_{\mathrm{s\_mic}}(q) = {} & N_{\mathrm{s}}{}^2\beta_{\mathrm{s}}{}^2 A_{\mathrm{s}}{}^2(q, R_{\mathrm{s}}) + N_{\mathrm{s}}\beta_{\mathrm{c}}{}^2 F_{\mathrm{c}}(q, R_{\mathrm{g}}) + N_{\mathrm{s}}(N_{\mathrm{s}} - 1)\beta_{\mathrm{c}}{}^2 A_{\mathrm{c}}{}^2(q) \\ & + 2N_{\mathrm{s}}{}^2\beta_{\mathrm{s}}\beta_{\mathrm{c}} A_{\mathrm{s}}(q, R_{\mathrm{s}}) A_{\mathrm{c}}(q) \end{aligned} \qquad S3$$

where R_{s} is the radius of the spherical micelle core, R_{g} is the radius of gyration of the PBeMA coronal block. The core block and the corona block X-ray scattering length contrast is given by $\beta_{\mathrm{s}} = V_{\mathrm{s}}(\xi_{\mathrm{s}} - \xi_{\mathrm{sol}})$ and $\beta_{\mathrm{c}} = V_{\mathrm{c}}(\xi_{\mathrm{c}} - \xi_{\mathrm{sol}})$, respectively. Here ξ_{s}, ξ_{c} and ξ_{sol} are the X-ray scattering length densities of the core block (ξ_{PBzMA} = 10.38 x 10^{10} cm^{-2}), the corona block (ξ_{PBeMA} = 9.26 x 10^{10} cm^{-2}) and the mineral oil solvent (ξ_{sol} = 7.63 x 10^{10} cm^{-2}), respectively.

V_s and V_c are volumes of the core block (V_{PBzMA}) and the corona block ($V_{PBeMA37}$), respectively. The volumes were obtained from $V = \frac{M_{n,pol}}{N_A \rho}$ using the density of PBzMA (ρ_{PBzMA} = 1.15 g cm^{-3})[2] and the solid-state homopolymer density of PBeMA determined by helium pycnometry (ρ_{PBeMA} = 0.97 g cm^{-3}), where $M_{n,pol}$ corresponds to the number-average molecular weight of the block determined by ^{1}H NMR spectroscopy. The scattering amplitude of the micelle core is expressed via sphere form factor amplitude:

$$A_c(q, R_s) = \Phi(qR_s)\exp\left(-\frac{q^2\sigma^2}{2}\right) \qquad S4$$

where $\Phi(qR_s) = \frac{3[\sin(qR_s) - qR_s\cos(qR_s)]}{(qR_s)^3}$. A sigmoidal interface between the two blocks was assumed for the spherical micelle form factor (Equation *S4*). This is described by the exponent term with a width σ accounting for a decaying scattering length density at the membrane surface. This σ value was fixed at 0.22 nm during fitting.

The scattering amplitude of the spherical micelle corona is:

$$A_c(q) = \frac{\int_{R_s}^{R_s+2s} \mu_c(r)\frac{\sin(qr)}{qr} r^2 dr}{\int_{R_s}^{R_s+2s} \mu_c(r) r^2 dr}\exp\left(-\frac{q^2\sigma^2}{2}\right) \qquad S5$$

The radial profile, $\mu_c(r)$, can be expressed by a linear combination of two cubic b splines, with two fitting parameters s and a corresponding to the width of the electron density corona profile and the function weight coefficient, respectively. This information can be found elsewhere,[3, 4] as can the approximate integrated form of Equation *S5*. The self-correlation term for the corona block is given by the Debye function:

$$F_\mathrm{c}(q,R_\mathrm{g}) = \frac{2\left[\exp\left(-q^2{R_\mathrm{g}}^2\right) - 1 + q^2{R_\mathrm{g}}^2\right]}{q^4{R_\mathrm{g}}^4} \qquad S6$$

where R_g is the radius of gyration of the PBeMA coronal block. The aggregation number of the spherical micelle is:

$$N_\mathrm{s} = (1 - x_\mathrm{sol})\frac{\frac{4}{3}\pi{R_\mathrm{s}}^3}{V_\mathrm{s}} \qquad S7$$

where x_sol is the volume fraction of solvent in the PBzMA micelle core. An effective structure factor expression proposed for interacting spherical micelles[5] has been used in Equation *S1*:

$$S_\mathrm{SF}(q) = 1 + \frac{A^\mathrm{av}_\mathrm{s\_mic}(q)^2[S(q) - 1]}{F_\mathrm{s\_mic}(q)} \qquad S8$$

Herein the scattering amplitude of the average radial scattering length density distribution of micelles is used as $A^\mathrm{av}_\mathrm{s\_mic}(q) = N_\mathrm{s}[\beta_\mathrm{s}A_\mathrm{s}(q,R_\mathrm{s}) + \beta_\mathrm{c}A_\mathrm{c}(q)]$ and $S_\mathrm{SF}(q)$ is either: (1) a hard-sphere interaction structure factor based on the Percus-Yevick approximation,[6] S_PY (q, D_PY, f_PY), where D_PY is the interaction distance and f_PY is the effective hard-sphere volume fraction or (2) a sticky hard-sphere interaction structure factor,[7] S_SHS (q, D_SHS, f_SHS, τ), where D_SHS is the interaction distance, f_SHS is the effective hard-sphere volume fraction and τ is the so-called 'stickiness parameter', which was fixed at 0.10 in all cases. A polydispersity of the micelle core radius (R_s) was incorporated in the structural model (Equation *S1*) assuming a Gaussian distribution:

$$\Psi(r_1) = \frac{1}{\sqrt{2\pi{\sigma_{Rs}}^2}}\exp\left(-\frac{(r_1 - R_\mathrm{s})^2}{2{\sigma_{Rs}}^2}\right) \qquad S9$$

where σ_{Rs} is the standard deviation for R_s. In accordance with Equation *S2* the number density per unit sample volume for the micelle model is expressed as:

$$N = \frac{\varphi}{\int_0^\infty V(r_1)\Psi(r_1)dr_1} \qquad S10$$

where φ is the volume fraction of copolymer in a sample and $V(r_1)$ is the total *volume* of copolymer in a spherical micelle $[V(r_1) = (V_s + V_c)N_s(r_1)]$.

The model fitting to the SAXS pattern of 1.0% w/w $PBeMA_{37}$-$PBzMA_{100}$ spheres at 50 °C indicated an experimental R_g for the corona PBeMA block equal to 1.49 nm, which is physically reasonable, since it lies close to a rough theoretical estimate for the parameter. Thus, assuming that the projected contour length of a PBeMA monomer is 0.255 nm (two C-C bonds in *all-trans* conformation), the total contour length of a $PBeMA_{37}$ block, $L_{PBeMA37}$ = 37 x 0.255 nm = 9.435 nm. Given a mean Kuhn length of 1.53 nm (based on the known literature value for PMMA[8]) an estimated unperturbed radius of gyration, R_g = (9.435 x 1.53/6)$^{0.5}$, or 1.55 nm is calculated.

An additional population represented by a power law dependence of scattering intensity (Bq^{-p}, where B is a constant and p is an exponent) was incorporated when fitting the SAXS pattern recorded at 20 °C. This approach enabled the upturn in scattering intensity observed at low q corresponding to large scattering objects formed by a micelle aggregation (Figures 7 and S2) to be approximated. It is reasonable to assume that the overall structural morphology can be described by mass fractals where p corresponds to the fractal dimension. Additionally,

a Gaussian peak ($A\exp\left[-\left(\frac{q-q_{\mathrm{peak}}}{width}\right)^2\right]$) was added to the model in order to account for the subtle feature observed at q ~ 1.8 nm^{-1} for the 20% w/w dispersion at 20 °C (Figure 7b, black data). Thus, the entire scattering pattern would be described as:

$$I(q) = \frac{d\Sigma}{d\Omega}(q) + Bq^{-p} + A\exp\left[-\left(\frac{q-q_{\mathrm{peak}}}{width}\right)^2\right] \qquad S11$$

where the first term represents scattering from spherical micelles (Equations *S1* and *S2*).

References

1. J. S. Pedersen, *Journal of Applied Crystallography*, 2000, **33**, 637-640.
2. L. A. Fielding, J. A. Lane, M. J. Derry, O. O. Mykhaylyk and S. P. Armes, *Journal of the American Chemical Society*, 2014, **136**, 5790-5798.
3. J. S. Pedersen and M. C. Gerstenberg, *Colloids and Surfaces a-Physicochemical and Engineering Aspects*, 2003, **213**, 175-187.
4. J. S. Pedersen, C. Svaneborg, K. Almdal, I. W. Hamley and R. N. Young, *Macromolecules*, 2003, **36**, 416-433.
5. J. S. Pedersen, *Journal of Chemical Physics*, 2001, **114**, 2839-2846.
6. D. J. Kinning and E. L. Thomas, *Macromolecules*, 1984, **17**, 1712-1718.
7. S. V. G. Menon, C. Manohar and K. S. Rao, *The Journal of Chemical Physics*, 1991, **95**, 9186-9190.
8. L. J. Fetters, D. J. Lohsey and R. H. Colby, in *Physical Properties of Polymers Handbook*, ed. J. E. Mark, Springer, New York, 2nd edn., 2007, ch. 25, pp. 447-454.
